# The Association Between Sitting Delivery During the Second Stage of Labor and Pelvic Floor Function in Primiparas: A Prospective Cohort Study

**DOI:** 10.3390/healthcare14121681

**Published:** 2026-06-12

**Authors:** Jingjing Gong, Bing Xie, Yuxuan Wei, Li Fu, Lili Xing, Xiaonan Liu, Xiaodan Li

**Affiliations:** 1 Department of Obstetrics and Gynecology, Peking University People’s Hospital, Beijing 100044, China; gongjingjing@pkuph.edu.cn (J.G.); bingxie@bjmu.edu.cn (B.X.); xinglili@pkuph.edu.cn (L.X.); liuxiaonan@pkuph.edu.cn (X.L.); 2 School of Nursing, Hebei University, Baoding 071002, China; 20248025002@stumail.hbu.edu.cn; 3 Nursing Department, Liaoning University of Traditional Chinese Medicine, Shenyang 110847, China; ful-hlxy@lnutcm.edu.cn

**Keywords:** sitting delivery, primiparas, second stage of labor, pelvic floor function

## Abstract

**Highlights:**

**What are the main findings?**

- Sitting delivery was associated with a higher rate of spontaneous vaginal delivery.
- Sitting delivery was associated with a shorter duration of the second stage of labor.

**What are the implications of the main findings?**

- It did not increase the incidence of urinary incontinence at one year postpartum.
- It did not increase the incidence of sexual dysfunction at one year postpartum.

**Abstract:**

Background/Objectives: Pregnancy and childbirth are independent risk factors for pelvic floor injury. The choice of delivery position, as an interventionable factor during labor, has received much attention. This study aimed to assess the impact of sitting delivery on pelvic floor function by observing the outcomes of the perineum, duration of the second stage of labor, integrity of pelvic floor structure, and postpartum pelvic floor function. The goal was to provide scientific evidence for optimizing delivery management and reducing the risk of pelvic floor injury. Methods: This was a non-randomized study (based on patient choice). A total of 222 primiparous women who delivered at our hospital from February to August 2023 were selected and divided into the control group (*n* = 88) and the sitting group (*n* = 134). The second stage of labor in the control group was delivered in the traditional bladder lithotomy position, while the sitting group delivered in a sitting position. The duration of the second stage of labor, delivery method, degree of perineal laceration, postpartum 2 h bleeding volume, and the occurrence of urinary incontinence and sexual dysfunction 1 year after delivery were observed in both groups. Results: The rate of perineal lateral incision in the sitting group was lower than that in the control group (24.6% vs. 48.9%, aOR = 0.37, *p* < 0.001); the adjusted mean difference in second-stage duration was −11.65 min (95% CI: −25.35 to 2.05, *p* = 0.095), indicating no statistically significant difference after adjustment. In unadjusted subgroup analysis, a larger point estimate for reduction was observed in women aged < 30 years (median difference −16.0 min, *p* = 0.033), but the interaction test was not significant. For urinary incontinence and sexual dysfunction, there were no statistically significant differences between the two groups. Conclusions: Sitting delivery was associated with a lower episiotomy rate and, in unadjusted analysis, a shorter second stage (although the adjusted difference was not significant), and it did not increase the incidence of urinary incontinence or sexual dysfunction at one year postpartum.

## 1. Introduction

Natural childbirth is one type of delivery method. The second stage of labor is an important part of natural childbirth and plays a crucial role in ensuring the health of both the mother and the newborn [1]. Free-position delivery refers to the position taken by the mother during labor, which is comfortable and can relieve pain, under the guidance of an obstetrician or midwife [2], including sitting position, knee-chest position, lateral position, upright position, etc. [3]. As an intervention factor during labor, the delivery position has received widespread attention in recent years. The World Health Organization [4] and the Canadian Association of Obstetricians and Gynecologists [5] have clearly encouraged free-position delivery during the second stage of labor.

Pregnancy and childbirth are well-established independent risk factors for pelvic floor dysfunction (PFD). During vaginal delivery, prolonged pressure and traction on the pelvic floor muscles, fascia, and nerves can result in anal sphincter tears, pudendal nerve injury, and stretching or degeneration of pelvic connective tissue [6]. Long-term consequences are substantial [7]: a cohort study reported that among women who had vaginal deliveries, 61% experienced urinary incontinence, 22% fecal incontinence, and 17% pelvic organ prolapse 20–26 years after delivery [8]. However, most existing studies on PFD have limitations, including cross-sectional designs, lack of adjustment for key confounders (e.g., parity, age, body mass index), and considerable population heterogeneity. For instance, the reported prevalence of PFD in postpartum women varies widely from 10% to 50% depending on the population studied, timing of assessment, and diagnostic criteria. A meta-analysis showed an average prevalence of 25.97%, with stress urinary incontinence accounting for 53.09% and sexual pain for 27.84% [9,10], but these estimates are derived predominantly from Western or mixed populations and may not be generalizable to other ethnic or obstetric groups.

Despite the increasing use of free positions, evidence on how specific positions affect postpartum pelvic floor function remains limited and inconclusive. Most studies have focused on short-term delivery outcomes (e.g., episiotomy rate, duration of the second stage, postpartum hemorrhage) and lack long-term follow-up of pelvic floor function [11,12,13]. In particular, the sitting delivery position—which takes advantage of gravity and allows better access for the attendant—has been gradually adopted in clinical practice, but there are also certain limitations: (1) increased maternal fatigue due to maintaining an upright posture, (2) possible higher risk of perineal lacerations, as suggested by some studies, (3) difficulty in performing episiotomy or operative vaginal delivery in the sitting position, (4) limited applicability in women with certain medical conditions (e.g., severe varicose veins, hip joint disorders), (5) the need for specialized delivery beds and trained staff. These potential drawbacks are often under-reported in the literature and warrant further investigation. And its effects on pelvic floor integrity and on the incidence of urinary incontinence or sexual dysfunction have not been systematically evaluated. Furthermore, previous studies rarely adjusted for important confounders such as maternal age, neonatal birth weight, and epidural analgesia, which may bias the association between delivery position and pelvic floor outcomes.

We hypothesized that sitting delivery would be associated with a lower episiotomy rate and a shorter second stage of labor, without increasing the risk of postpartum urinary incontinence or sexual dysfunction at one year after delivery. Exploratorily, we also examined whether the associations differed by maternal age and neonatal birth weight, though the primary hypothesis focused on the overall effects. The aim of this study is to assess the impact of sitting delivery on perineal outcomes, labor duration, and postpartum pelvic floor function, thereby providing scientific evidence for optimizing delivery management and reducing the risk of pelvic floor injury.

## 2. Materials and Methods

A total of 222 normal primiparous women who were admitted to a tertiary general hospital in Beijing from February to August 2023 were selected as the research subjects. Inclusion criteria: ➀ gestational age of 37 to 42 weeks, singleton pregnancy, cephalic presentation, occipital presentation; ➁ normal fetal heart rate and position as confirmed by prenatal examination; ➂ normal pelvic measurement and meeting the indications for vaginal delivery; ➃ regular prenatal check-ups at this hospital. Exclusion criteria: ➀ multiparous women; ➁ pregnant with severe complications; ➂ predicted neonatal birth weight less than 2500 g or more than 4000 g; ➃ with anal fissure, anal fistula or history of rectal surgery; ⑤ communication disorders or mental illness. Exclusion criteria: During the labor process, if there was fetal distress or other situations requiring shortening the labor process (such as fever during delivery, suspected intrauterine infection, etc.). Finally, 222 cases were included in the study. Allocation to delivery position was based on maternal preference after standardized counseling by midwives. Both positions (sitting and lithotomy) were explained to all eligible women during the first stage of labor. Women who expressed a clear preference for sitting delivery were assigned to the sitting group (*n* = 134); those who preferred or accepted the traditional lithotomy position were assigned to the control group (*n* = 88). The continuous sampling method was adopted, and no random grouping was conducted.

All deliveries were attended by midwives with ≥3 years of experience, and the same team rotated between both positions to reduce operator bias. Both groups of parturients received guidance from midwives during the first stage of labor and received necessary labor monitoring and routine care. They were placed in a free position for waiting, and the parturients were encouraged to perform free position activities as long as their physical condition permitted, including walking, sitting, standing, lying, and a series of activities. The parturients could choose the most comfortable and suitable position for waiting. ① Control group: During the second stage of labor, the parturients in the control group adopted the lithotomy position until the fetus and placenta were completely delivered. During the delivery process, the parturients could slightly raise their heads, bend and extend their legs, and place their feet on the supports on both sides of the delivery bed. During contractions, abdominal deep breathing was used, and the parturients were instructed to actively hold their breath and exert downward force. The parturients could grip the handrails on the bed for support during contractions and rest and relax during the intervals. After the fetal head crowned, the parturients were instructed to perform the “inhale” action. During the intervals, the parturients slowly delivered the fetal head and body. After the birth of the newborn, the newborn was placed on the parturient’s body for immediate skin contact, and routine care such as warmth was provided by the assistant at the table. The assisting midwife placed a blood collection device under the parturient’s buttocks to collect vaginal bleeding within 1 min, collected umbilical artery blood gas during the umbilical cord ligation, and then continued to delay the umbilical cord ligation. The placenta was delivered using the conventional method, and the bleeding and soft birth canal were checked. ② Observation group: During the waiting process, the midwife explained the benefits of sitting delivery, the cooperation methods again, and obtained the consent of the parturient and her family. During the second stage of labor, the angle of the delivery bed was adjusted to about 90°, allowing the parturient to sit on the bed. A soft pillow was placed at the gap between the parturient’s waist and the bed, and the bed head assisted in supporting the parturient’s upper body. The parturient’s feet were placed on the pedals. During the adjustment of the position, the parturient was encouraged to keep their arms, thighs, and trunk relaxed, have smooth breathing, and focus their attention. If the parturient felt autonomous force during contractions, they were encouraged to wait until the strongest contractions occurred before starting to exert force. During the forceful exertion, the parturient quickly inhaled through the nose and slowly exhaled through the mouth, concentrating on the slow exhalation movement. During exhalation, the transverse diaphragm and rectus abdominis muscles of the parturient exerted pressure on the uterus (abdominal cavity), and the midwife assisted the parturient to exert downward force on the fetus. The fetus gradually descended under the combined action of uterine contractions, the parturient’s transverse diaphragm and rectus abdominis muscles, gravity, and multiple forces. During the intervals, the parturient was instructed to relax the whole body and adjust to normal breathing. When the fetal head was 2–3 cm above the pubic symphysis, the parturient was gradually relaxed and adjusted to normal breathing. The fetus continued to descend under the action of uterine contractions and gravity. When the fetal occipital bone reached below the pubic symphysis, it was resisted by the levator ani muscle, and began to gradually extend. The midwife assisted the parturient to slowly slide out of the vaginal opening. After the fetus was delivered, the newborn was placed in the parturient’s arms for immediate skin contact, and the assistant at the table performed routine operations such as warmth for the newborn. The midwife placed a blood collection device under the parturient’s buttocks to collect vaginal bleeding, completed the delayed umbilical cord ligation and other operations. Evaluation method: ➀ Duration of the second stage of labor. ➁ Degree of perineal laceration, rate of perineal episiotomy and Postpartum hemorrhage (blood loss ≥ 500 mL within 24 h postpartum). ➂ Natural delivery rate (spontaneous vaginal delivery without forceps or vacuum). ➃ Occurrence of postpartum urinary incontinence: A 1-year follow-up call was conducted, and the degree of urinary incontinence of the parturients was understood using the International Consultation on Incontinence Questionnaire—Short Form (ICI-Q-SF) [11]. This scale has four dimensions, including urine leakage volume, frequency of leakage, time of leakage occurrence, and impact on daily life. The total score ranges from 0 to 21, with a higher score indicating a more severe degree of urinary incontinence. ➄ Postpartum sexual function: The Pelvic Floor/Urinary Incontinence Sexual Function Questionnaire Short Form (PISQ-12) [12] was used, consisting of 12 subjective questions, including three dimensions: emotional factors (sexual desire, frequency of sexual life, intensity and frequency of reaching orgasm), physiological factors (the degree of impact of urinary incontinence on sexual life, including whether there is urine leakage during sexual life and the resulting negative emotions), and partner factors (whether the sexual partner has premature ejaculation and erectile dysfunction and the acceptance level of urinary incontinence or pelvic floor disorders). The higher the score, the higher the quality of sexual life. Follow-up and missing data: During the 1-year follow-up period, all 222 pregnant women completed the ICI-Q-SF and PISQ-12 questionnaires. During the 1-year follow-up period, all 222 women completed the ICI-Q-SF and PISQ-12 questionnaires at 12 months postpartum. Follow-up was conducted via telephone interviews. For participants not reachable after three telephone attempts, their family members were contacted to facilitate completion. Using this approach, no participants were lost to follow-up (retention rate: 100%). However, we acknowledge that a 100% retention rate, while achieved, does not eliminate the risk of non-response bias or social desirability bias, particularly for sensitive outcomes (see Limitations). This study was registered with the Chinese Clinical Trial Registry (ChiCTR) on 18 April 2022 under the registration number ChiCTR2200058863. The full registry title is “Application and preliminary evaluation of the Practice Programme for Upright Positions in the Second Stage of Labour”. All procedures were conducted in accordance with the principles of the Declaration of Helsinki, and has been approved by the Institutional Review Board for Ethics (2022PHB189-001).

### Statistical Methods

Data were analyzed using R (4.5.1). Continuous variables following a normal distribution were expressed as mean and standard deviation, and compared using the independent sample *t*-test, including maternal age, BMI during pregnancy, gestational weeks, and neonatal weight. Continuous variables not following a normal distribution were expressed as median and interquartile range, and compared using the Mann–Whitney U test, including the number of pregnancies. Categorical variables were expressed as frequencies and percentages, and compared using Fisher’s exact test, including educational level. To comprehensively evaluate the duration of the second stage of labor, three complementary analytical approaches were employed. The primary analysis utilized a multivariable linear regression model to calculate the adjusted Mean Difference (aMD), controlling for predefined clinical covariates, including maternal age, BMI, gestational weeks, parity and neonatal weight, to isolate the independent effect of the sitting position. Secondarily, a time-to-event analysis using the Kaplan–Meier method and the Log-rank test was conducted to evaluate the dynamic probability of delivery over time, capturing the temporal acceleration that might be masked by extreme outliers in mean comparisons. Finally, subgroup analyses employing unadjusted linear regression within specific strata were performed to explore specific populations benefiting most from the intervention. For other dichotomous delivery outcomes, such as episiotomy and perineal lacerations, multivariate Logistic regression analysis was used to calculate adjusted odds ratios (aOR). A two-sided *p*-value < 0.05 was considered statistically significant. A post hoc power analysis was conducted to assess the sufficiency of the sample size in the case of unbalanced groups. Based on the primary binary outcome, using the actual sample sizes of the seated group (*n* = 134) and the control group (*n* = 88), the statistical power (1 -β) was calculated assuming a two-sided α of 0.05.

## 3. Results

### 3.1. Patient Characteristics

The comparison of age, educational level, gestational weeks, maternal BMI during pregnancy, and neonatal birth weight between the two groups is shown in Table 1.

### 3.2. Comparison of Perineal Integrity

**Figure 1:**
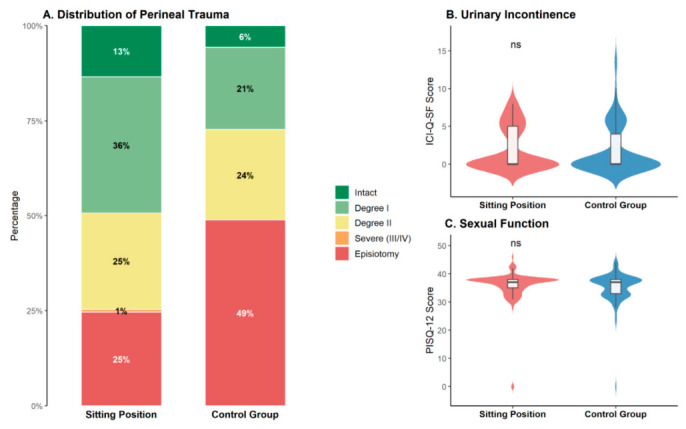
Comparison Chart of Perineal Injury Severity and Long-Term Safety. Caption: (**A**) The stacked bar chart shows the percentage distribution of different degrees of perineal injury. The colors represent the injury types: green = intact perineum, light green = grade I laceration, yellow = grade II laceration, orange = severe laceration (grade III/IV), red = perineal episiotomy. The episiotomy rate in the sitting group was significantly lower, but the proportion of mild lacerations increased. (**B**) The violin plot of the postpartum urinary incontinence score (ICI-Q-SF). (**C**) The violin plot of the postpartum sexual function score (PISQ-12). The box plots within the violin plots show the median and interquartile range. ns indicates no statistically significant difference between the two groups (*p* > 0.05).

Multivariate logistic regression analysis was used to analyze the delivery outcomes of the sitting delivery group and the control group. The statistical results showed significant differences between the two groups in terms of natural delivery rate, episiotomy rate, and overall perineal laceration rate (*p* < 0.05). The sitting group had a significantly higher natural delivery rate than the control group. The possibility of natural delivery for the sitting group was 3.65 times that of the control group (aOR = 3.65, 95% CI: 1.68–8.34, *p* = 0.001); there was no statistical difference in the duration of the second stage of labor between the two groups; in terms of perineal laceration, the sitting group significantly reduced the intervention rate of episiotomy (24.6% vs. 48.9%, aOR = 0.37, *p* < 0.001). Although the overall rate of perineal laceration in the sitting group was statistically higher than that in the control group (61.9% vs. 45.5%, aOR = 1.89, *p* = 0.027), further stratified analysis revealed that this increase was mainly concentrated in grade I perineal laceration (*p* = 0.040), while there was no significant difference in grade II (*p* = 0.775) and severe grade III/IV lacerations between the two groups. The stacked bar chart in Figure 1A also visually presents this result; in terms of postpartum hemorrhage (≥500 mL), there was no statistical difference between the two groups (*p* > 0.05) (Table 2).

**Table 2:** Results of Delivery Outcomes and Multivariate Logistic Regression Analysis.

	Sitting Group	Control Group	Adjusted Effect Measure(95% CI)	*p* Value
Natural childbirth	122 (91%)	64 (72.7%)	3.65 (1.68–8.34)	0.001
Duration of the second stage (min), Median [IQR]	55.0 [35.0–80.2]	73.0 [47.8–105.8]	−11.65 (−25.35–2.05)	0.095 *
Perineal lateral incision	33 (24.6%)	43 (48.9%)	0.37 (0.20–0.67)	<0.001
Total perineal laceration	83 (61.9%)	40 (45.5%)	1.89 (1.08–3.32)	0.027
I degree laceration	48 (35.8%)	19 (21.6%)	1.97 (1.04–3.82)	0.040
II degree laceration	34 (25.4%)	21 (23.9%)	1.10 (0.58–2.11)	0.775
III degree laceration	1 (0.7%)	0 (0%)	-	-
IV degree laceration	0 (0%)	0 (0%)	-	-
Postpartum hemorrhage (≥500 mL)	12 (9%)	7 (8%)	1.29 (0.47–3.75)	0.628

**Figure 2:**
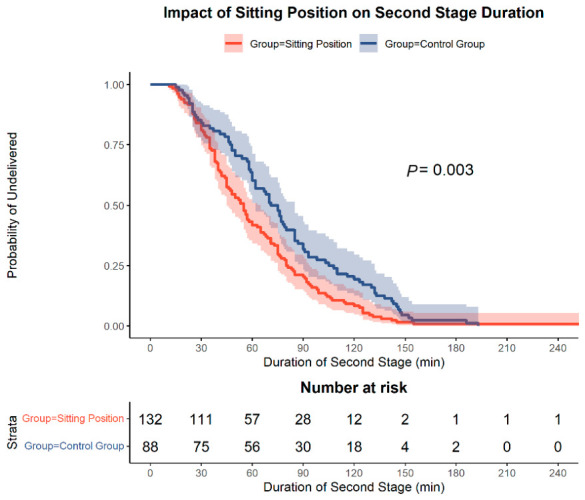
Kaplan–Meier survival curve for the duration of the second stage of labor. Caption: The red curve represents the group with sitting delivery, while the blue curve represents the control group. The x-axis indicates the duration of the second stage of labor (in minutes), and the y-axis represents the probability of not delivering. The Log-rank test showed that the unadjusted second-stage duration was significantly shorter in the sitting group than in the control group (*p* = 0.003). Below the chart, the number of individuals at risk at each time point is listed.

* Note: The Mann–Whitney U test p = 0.095; the Kaplan–Meier log-rank test p = 0.003 (see Figure 2). Adjusted effect size: For categorical outcomes, the effect size is the aOR derived from multivariate logistic regression; for continuous outcomes (duration of the second stage of labor), although the original data has a right-skewed distribution and the median [IQR] is used for description, the effect size is the adjusted mean difference (aMD) derived from multivariate linear regression. All models have accounted for factors such as gestational age, gestational weeks, parity, neonatal weight, and labor analgesia.

### 3.3. Comparison of Pelvic Floor Tissue Ischemia/Tension Time

#### 3.3.1. Comparison of the Duration of the Second Stage of Labor

The Kaplan–Meier method was used to compare the second-stage duration between the two groups of parturients. The results showed that there was a significant statistical difference in the duration of the second stage of labor between the sitting delivery group and the control group (Log-rank test, *p* = 0.003). As time progressed, the “probability of not delivering” curve of the sitting group declined at a significantly faster rate than that of the control group, indicating that the proportion of parturients in the sitting group who completed delivery within the same time frame was higher. Especially in the interval from 60 to 120 min of the labor process, the separation of the two curves was the most obvious. (Figure 2).

#### 3.3.2. Subgroup Analysis of the Duration of the Second Stage of Labor

**Figure 3:**
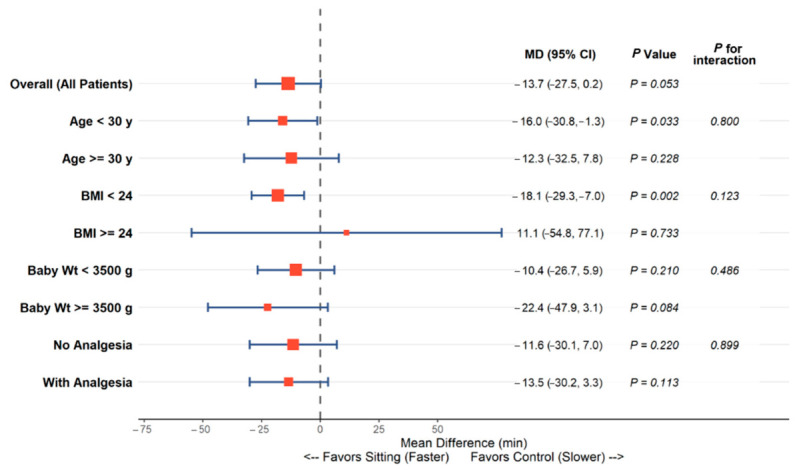
Forest plot of subgroup analysis of the duration of the second stage of labor. Caption: The red squares in the figure represent the mean difference, and the blue horizontal lines represent the 95% confidence interval (95% CI). The vertical dotted line (0 point) is the null line. A negative MD value (on the left side of the dotted line) indicates that the labor process in the sitting delivery group is shorter, while a positive value (on the right side of the dotted line) indicates that the labor process in the control group is shorter. The unadjusted point estimates suggested a significant reduction in the sitting group for the age < 30 and BMI < 24 subgroups (*p* < 0.05 for each), but the formal interaction tests were not significant (*p* for interaction > 0.05). On the right side of the figure, the *p* values for the interaction calculated from each subgroup variable are presented (*p* for interaction).

The unadjusted data show that the mean second-stage duration in the sitting group was 13.7 min shorter than in the control group, a difference that was on the verge of statistical significance (MD = −13.7, 95% CI: (−27.5, 0.2), *p* = 0.053). Further examination of the subgroups revealed: First, for young women (age < 30 years), the sitting group had a significantly shorter second stage of labor by 16.0 min (MD = −16.0, *p* = 0.033), while for parturients aged 30 years and above, although there was a trend of shortening, the difference was not statistically significant. Second, for parturients with low/normal body mass index (BMI < 24), the sitting group had an average reduction of 18.1 min in the second stage of labor (MD = −18.1, *p* = 0.002). In the overweight/obese group (BMI ≥ 24), the confidence interval was extremely wide, suggesting unstable effects. Third, regardless of whether the fetal weight was <3500 g or ≥500 g, although the trend favored the sitting group for a faster second stage of labor, the confidence intervals crossed the 0 line, indicating no statistically significant difference (Figure 3).

The exploratory subgroup analysis revealed a potential trend: younger mothers and those with low/normal gestational ages may benefit more significantly from sitting delivery in shortening the labor process. However, it should be noted that the formal interaction test did not reach statistical significance. Therefore, the differences in these subgroups lack formal statistical support for heterogeneity and must be interpreted cautiously as exploratory findings. Further verification is needed in larger-scale trials.

### 3.4. Comparison of Urinary Incontinence Incidence Rates

The median and interquartile range of ICI-Q-SF scores for the sitting group and the control group were basically the same, and there was no statistically significant difference (ns) (Figure 1B).

### 3.5. Comparison of the Incidence of Sexual Dysfunction

In the PISQ-12 score, the distribution density of the data from both groups mainly concentrated in the higher segments, and there was no statistically significant difference (ns) (Figure 1C).

### 3.6. Adverse Events Reporting the Following

No maternal deaths or ICU admissions occurred. No grade IV perineal lacerations were observed; one grade III laceration occurred in the sitting group. Postpartum hemorrhage (≥500 mL) occurred in 12 (9.0%) vs. 7 (8.0%), not significant. Neonatal outcomes: Apgar scores at 1 and 5 min were similar between groups (all ≥ 8). No neonatal fractures or brachial plexus injuries.

## 4. Discussion

The following discussion interprets the observed associations. Given the observational design, no causal or mechanistic claims can be made.

### 4.1. Sitting Delivery Was Associated with Enhanced Perineal Integrity and a Lower Episiotomy Rate

The results of this study show that the sitting group had a significantly lower rate of perineal episiotomy compared to the control group (24.6% vs. 48.9%, aOR = 0.37, *p* < 0.001). This finding is consistent with previous studies [13]. A possible explanation, though speculative, is that sitting delivery more closely mimics daily defecation posture, allowing the backrest and armrest to support the lumbar and upper body, potentially reducing perineal tension and facilitating relaxation of the pelvic floor muscles [14]. However, our study did not directly measure pelvic floor muscle activity or continuity; therefore, these mechanistic interpretations remain hypothetical and require confirmation in future studies using objective measures.

A key trade-off was observed: sitting delivery reduced episiotomy (24.6% vs. 48.9%) but was associated with a higher overall rate of perineal laceration, Severe perineal lacerations can cause serious adverse effects on the parturient [15]. he results of this study show that primarily grade I (35.8% vs. 21.6%). Grade I lacerations are superficial, typically heal without long-term sequelae, and are not associated with an increased risk of pelvic floor dysfunction. In contrast, episiotomy is a surgical incision that may cause more pain, blood loss, and postpartum dyspareunia. The observed pattern—fewer episiotomies but more grade I lacerations in the sitting group—may be considered favorable by some clinicians, but we emphasize that our study did not directly compare patient-reported outcomes such as perineal pain, healing satisfaction, or preference. Therefore, we refrain from labeling this pattern as “acceptable” or “unacceptable” without patient-centered data.

### 4.2. Association Between Sitting Delivery and Duration of the Second Stage of Labor

As shown in Figure 1, over time, the “probability of not delivering” curve of the sitting group decreased significantly faster than that of the control group, indicating that the proportion of completing delivery in the sitting group was higher within the same period. Especially in the 60 to 120 min interval of the labor progress, the separation of the two groups’ curves was the most obvious, suggesting a temporal association between sitting position and a shorter second stage, consistent with the reports in the related literature [16,17]. The possible reason is that sitting delivery changes the relationship between the pelvis and the fetal longitudinal axis during the second stage of labor by changing the body position, increasing the transverse diameter of the pelvic middle plane and the pelvic outlet plane, and expanding the pelvic space [18,19]. In addition, the spine and pelvis form a C-shaped channel, making it easier for the infant’s head to rotate and descend. At the same time, the effect of gravity can effectively promote the progress of labor and shorten the duration of the second stage of labor. On the other hand, during free position delivery, the pressure during the static period of the uterus increases, and the intrauterine pressure during the intercontraction period is higher, which can promote the descent of the fetal presenting part, thereby promoting the progress of labor. Moreover, this study suggests that sitting delivery has a more significant reduction in the second stage of labor for primiparas under the age of 30 and for primiparas with a BMI of less than 24, regardless of the size of the fetal weight and whether labor analgesia is used. The reason is that during sitting delivery, the abdominal and pelvic floor muscles contract more strongly, the sacrococcygeal joint is not compressed, and it is easier to expand; in addition, during sitting delivery, the trunk is perpendicular to the ground, which can fully utilize the effective space within the birth canal and the gravitational and centripetal forces of the fetus, accelerating the descent of the fetus, a shorter second stage may theoretically reduce the duration of mechanical load on the pelvic floor [20], which could be beneficial, but our study did not directly assess anal sphincter integrity, nerve function, or ischemia–reperfusion injury. Therefore, we cannot conclude that sitting delivery “protects” the pelvic floor. These physiological hypotheses should be tested in future studies using objective assessments such as endoanal ultrasound, perineal nerve conduction studies, or pelvic floor MRI.

### 4.3. Association Between Sitting Delivery and Natural Delivery Rate

In recent years, safety of delivery and management during labor have been the most concerned issues in the delivery process. Effective position management can promote natural delivery and reduce medical expenses [21]. The sitting group had a higher rate of natural delivery (91.0% vs. 72.7%, aOR = 3.65, *p* = 0.001). This finding is consistent with previous studies [16,22,23,24]. Analysis of the reasons for the increase in the rate of natural childbirth: Abnormal fetal position during the second stage of labor is one of the important reasons leading to difficult labor or an increase in the rate of cesarean section transfer. When in a sitting position, it can increase the pelvic outlet area and change the pelvic inclination, correct the occipito-transverse position and occipito-posterior position, and promote natural childbirth [14]. The results of this study show that sitting delivery did not increase the volume of bleeding at 2 h postpartum, consistent with Dénakpo et al. [25]. This may be because the same preventive measures, such as using oxytocin to promote uterine contractions, were taken in the first stage of labor and immediately after the fetus was delivered, which effectively reduced the amount of bleeding 2 h after delivery in both groups of women. In addition, the comfort of the mother during childbirth was improved, and the incidence of postpartum uterine atony and soft birth canal laceration was almost zero, thereby reducing the amount of postpartum bleeding. Therefore, there was little difference in the amount of bleeding 2 h after delivery between the two groups. One possible explanation, though speculative, is that the sitting position may increase the pelvic outlet area and correct malpositions, but our study did not assess fetal position changes directly. Importantly, we cannot conclude that sitting delivery “avoids” anal sphincter tears or nerve injury, as we did not perform endoanal ultrasound or nerve conduction studies.

### 4.4. Sitting Delivery Does Not Increase the Incidence of Postpartum Urinary Incontinence

The International Continence Society (ICS) defines urinary incontinence as the process of involuntary urine flow from the external urethral orifice under any circumstances, which can be divided into three types, including stress urinary incontinence (SUI), urge urinary incontinence (UUI), and mixed urinary incontinence (MUI). The most common type is SUI [26,27]. It is the most common type of pelvic floor dysfunction (PFD) and is caused by defects or injuries to the pelvic floor support tissues. The World Health Organization defines postpartum SUI as the phenomenon related to pregnancy and childbirth that occurs after delivery, that is, when actions such as sneezing, coughing, laughing, or movement that increase abdominal pressure occur, the patient’s urine involuntarily flows from the external urethral orifice [28]. The pathogenesis of SUI is closely related to the changes in pelvic–abdominal dynamics during pregnancy and the injury during childbirth [29]. During childbirth, excessive dilation of the birth canal causes the levator ani muscle to tear, and for those with a second stage of labor lasting more than 3 h, the elastic modulus of the pelvic floor fascia decreases, forming a persistent “suspended structure” dysfunction, ultimately leading to a decrease in urethral closure pressure (<30 cm H_2_O), and thereby causing SUI. Pregnancy and childbirth are usually considered independent risk factors for SUI, and Danforth et al. [30] proposed that the prevalence of SUI in American first-time parturient women is 9.7%, which seriously affects the quality of life of patients [31]. The results of this study show that there is no statistically significant difference in the incidence of urinary incontinence 1 year after childbirth between the two groups of women (*p* > 0.05), suggesting that sitting delivery was not associated with an increased incidence of urinary incontinence.

### 4.5. Sitting Delivery Does Not Increase the Incidence of Maternal Sexual Dysfunction

Female sexual dysfunction (FSD) refers to functional disorders in the female sexual response cycle, which may manifest as problems in the female’s sexual arousal, sexual desire, sexual climax, or sexual pain during sexual intercourse. FSD seriously hinders the experience of a certain or multiple stages of the female sexual response cycle, significantly is associated with a reduction in the quality of her sexual life, and may have serious adverse effects on her physical and mental health [32]. During vaginal delivery, muscle fibers are in a state of continuous tension, the vaginal wall is reshaped, muscle fatigue increases, or there are different degrees of tears, all of which can lead to an increase in the incidence of FSD [33]. In this study, there was no statistically significant difference in the incidence of postpartum sexual dysfunction between the two groups of women who delivered in different positions (*p* > 0.05), which suggests that sitting delivery was not associated with a higher incidence of sexual dysfunction at one year postpartum. However, sexual function is multifactorial, and our study did not assess vaginal anatomy, lubrication, or dyspareunia directly.

### 4.6. Appraising Randomized Evidence

Several randomized trials have compared upright vs. supine positions (e.g., Moraloglu 2017 [16], Simarro 2017 [17], and the Cochrane review by Gupta et al. 2017 [23]). However, these trials often had small sample sizes, unclear allocation concealment, and variable definitions of outcomes. The Cochrane review concluded that the evidence for upright positions is low to moderate quality. Our observational study cannot replace randomized evidence but adds real-world data on long-term pelvic floor outcomes, which are lacking in most trials.

### 4.7. Limitations

This study has several limitations. First, the non-randomized allocation based on maternal preference may introduce selection bias and unmeasured confounding (e.g., motivation, pain tolerance). Second, the sample size was modest (*n* = 222) with imbalanced groups, although post hoc power analysis indicated sufficient power for the primary outcomes (episiotomy and natural delivery rates). Third, the single-center, homogeneous sample (urban, highly educated primiparas) limits generalizability. Fourth, pelvic floor function was assessed only by self-report questionnaires (ICI-Q-SF, PISQ-12); we did not perform objective measurements (ultrasound, perineometry, EMG). Fifth, although we achieved 100% follow-up, the use of telephone interviews may introduce social desirability bias and recall bias. Sixth, outcome assessors were not blinded to delivery position. Finally, the discrepancy between unadjusted KM and adjusted regression results for second-stage duration highlights the sensitivity of conclusions to statistical methods. These limitations preclude causal inference.

## 5. Conclusions

In this prospective cohort study of 222 primiparous women, sitting delivery during the second stage of labor was associated with a significantly lower episiotomy rate and no statistically significant increase in urinary incontinence or sexual dysfunction at one year postpartum. The adjusted difference in second-stage duration was not significant, although an unadjusted Kaplan–Meier analysis suggested faster delivery in the sitting group. Subgroup analyses by age and birth weight were exploratory; interaction tests were not significant. Therefore, no age- or weight-specific clinical recommendations can be made. Given the non-randomized design and potential residual confounding, these findings should be considered hypothesis-generating. Randomized controlled trials with objective pelvic floor assessments are needed to confirm the observed associations.

## Figures and Tables

**Table 1:** Comparison of General Data between the Two Groups (*n* = 222).

Project	Sitting Group (*n* = 134)	Control Group (*n* = 88)	Total (*n* = 222)	*p*
Age (Mean ± SD)	30.58 ± 2.79	30.59 ± 2.79	30.59 ± 2.79	0.982
Gestational weeks (Mean ± SD)	39.26 ± 1.08	39.34 ± 1.00	39.29 ± 1.05	0.581
Maternal BMI during pregnancy (Mean ± SD)	21.19 ± 2.83	21.68 ± 2.87	21.38 ± 2.85	0.203
Newborn birth weight (g) (Mean ± SD)	3203.34 ± 331.60	3172.03 ± 332.19	3251.02 ± 326.82	0.083
Duration of the first stage of labor (Median [IQR])	410.00 [290.00, 525.00]	420.00 [300.00, 652.50]	20.00 [290.00, 560.00]	0.142
Number of pregnancies (Median [IQR])	1.00 [1.00, 1.00]	1.00 [1.00, 1.00]	1.00 [1.00, 1.00]	0.504
Educational attainment(%)				0.460
High school and below	0 (0.0%)	1 (1.1%)	1 (0.5%)	
Associate degree/undergraduate degree	88 (65.7%)	58 (65.9%)	146 (65.7%)	
Postgraduate degree and above	46 (34.3%)	29 (33.0%)	75 (33.8%)	
Whether to use labor analgesia				0.315
No	86 (64.2%)	63 (71.6%)	149 (67.1%)	
Yes	48 (35.8%)	25 (28.4%)	73 (32.9%)	

Note: Continuous variables that follow a normal distribution are expressed as mean (standard deviation), and independent sample *t*-test was used for age, maternal BMI during pregnancy, gestational weeks, and neonatal weight; continuous variables that do not follow a normal distribution are expressed as median [interquartile range], and the Mann–Whitney U test is used (including the number of pregnancies); categorical variables are expressed as frequency (percentage), and Fisher’s exact test (including educational attainment) is used to calculate *p* value.

## Data Availability

The data that support the findings of this study are available from the corresponding author.
